# Etiological Insights and the Role of Individual Factors in Infectious Spondylodiscitis

**DOI:** 10.3390/idr17010006

**Published:** 2025-01-10

**Authors:** Diana Elena Vulpe, Dana-Georgiana Nedelea, Serban Dragosloveanu, Oana Sandulescu, Cristian Scheau

**Affiliations:** 1 Doctoral School, The “Carol Davila” University of Medicine and Pharmacy, 050474 Bucharest, Romania; diana-elena.plescan@drd.umfcd.ro; 2 Department of Orthopaedics, “Foisor” Clinical Hospital of Orthopaedics, Traumatology and Osteoarticular TB, 021382 Bucharest, Romania; dg_nedelea@yahoo.com; 3 Department of Orthopaedics and Traumatology, The “Carol Davila” University of Medicine and Pharmacy, 050474 Bucharest, Romania; 4 Department of Infectious Diseases I, Faculty of Medicine, Carol Davila University of Medicine and Pharmacy, 050474 Bucharest, Romania; oana.sandulescu@umfcd.ro; 5 National Institute for Infectious Diseases “Prof. Dr. Matei Bals”, 021105 Bucharest, Romania; 6 Academy of Romanian Scientists, 050044 Bucharest, Romania; 7 Department of Physiology, The “Carol Davila” University of Medicine and Pharmacy, 8 Eroii Sanitari Boulevard, 050474 Bucharest, Romania; cristian.scheau@umfcd.ro; 8 Department of Radiology and Medical Imaging, “Foisor” Clinical Hospital of Orthopaedics, Traumatology and Osteoarticular TB, 021382 Bucharest, Romania

**Keywords:** spondylodiscitis, spinal infection, microbiological analyses, microorganisms, comorbidities, mycobacterium tuberculosis, physiopathology

## Abstract

**Objectives:** Spondylodiscitis can be caused by various microorganisms and has shown a continuous rise in incidence and mortality. The purpose of our study was to analyze the demographic and laboratory data, as well as comorbidities of patients that were surgically treated for spondylodiscitis in our hospital. The causative pathogens involved in the etiology of spinal infections were also assessed. **Methods:** The study included 92 patients who underwent clinical, radiological, and microbiological analyses including bacterial isolation. According to their culture results, patients were divided into three groups: negative results (*n =* 29), positive results with *Mycobacterium tuberculosis* (M. tb.) (*n =* 26), and positive results with other pathological agents (*n =* 37). **Results:** Patients with M. tb. had a significantly lower body mass index (*p* = 0.022) and were significantly younger (*p* = 0.024) than the others. The analysis of the complete blood work showed significant differences between the groups regarding fibrinogen levels (*p* = 0.023), C-reactive protein (*p* = 0.009), and erythrocyte sedimentation rates (*p* = 0.042). Results also showed significant differences (*p* = 0.023) for patients with diabetes mellitus who were more prone to a tuberculosis etiology for their spondylodiscitis compared with patients without the disease. **Conclusions:** These findings have important implications for adopting individualized treatment strategies underlining the need for identification of patients at high risk for specific causative pathogens.

## 1. Introduction

Spondylodiscitis is an infection that affects the intervertebral disc and the adjacent vertebrae and can be caused by numerous various organisms. The commonly involved pathogens in the development of spondylodiscitis are most frequently bacteria, causing a pyogenic spondylodiscitis, but *Mycobacterium tuberculosis* (M.tb.) and *Brucella* along with fungi and parasites can also represent the etiological cause for spinal infections, leading to granulomatous spondylodiscitis [1].

Spondylodiscitis is a rare disease, with an estimated incidence of around 2–7% of all cases of musculoskeletal infections [2]. The incidence is rising, probably due to the aging of the general population, with higher numbers of older people with chronic comorbidities, immunosuppressive therapies, or immunocompromised states, and the application of more sensitive and efficient diagnostic methods [3]. Mortality rates are between 2–4% and up to 15% in severe cases, depending on the pathogen involved, the affected segment of the spine, and other conditions related to the general health status of the patient [4,5,6]. Previous surgery for decompression, prior instrumentation and drainage, as well as spinal malformations represent additional risk factors for discitis [7,8,9].

A worldwide health problem, spondylodiscitis may require aggressive treatment that can carry extremely high economic costs, significant mortality, and antibiotic-resistance issues due to incorrect or incomplete treatment. Most cases of spondylodiscitis result from hematogenous spread from a different infectious site [10]. Predisposing factors known to influence the high prevalence of spinal infection are immunosuppressive conditions, diabetes mellitus, cancer, malnourishing conditions such as alcoholism, intravenous drug use, rheumatic or other immunological disease, renal failure and dialysis, hepatic failure, and previous spinal interventions [11,12]. Diagnosis of spondylodiscitis is based on non-specific symptoms related to the level of the spine affected, laboratory assessment, radiological and imaging examinations, and biopsy with tissue sampling. The low specificity of the signs and symptoms of spondylodiscitis usually delays the patient presentation to 2 to 6 months after the first symptoms [13]. The most frequent complaint is worsening pain in the affected spine region associated with spasms of the paraspinal musculature. Neurological involvement may occur from the start or during disease progression. Non-specific symptoms of infection such as fever, malaise, night sweats, or weight loss can be present [14,15].

Radiography, Computed Tomography (CT), and Magnetic Resonance Imaging (MRI) are useful imaging techniques for evaluating the presence of infection, the progressive structural changes within the affected spinal region, as well as the response to treatment. However, these imaging techniques have low specificity in providing information about the etiological pathogen [16,17,18,19].

Laboratory studies will generally show a normal or elevated white blood cell (WBC) count along with increased C-reactive protein (CRP) levels and Westergren erythrocyte sedimentation rate (ESR). Anemia, as well as low albumin levels, may be present, being a factor risk for sepsis [20]. Identification of the pathological agent can be extremely difficult in some situations, but correct and complete identification of the microorganism along with determination of the antibiotic sensitivity will improve patient outcomes [21]. Microbiological analyses and pathogen cultures should be obtained whenever possible. Pathogen cultures from guided biopsies or tissue sampling, along with newer techniques of molecular or PCR techniques can increase the diagnostic sensitivity and therapeutic efficiency [22].

The most frequent microorganism incriminated in the etiology of spondylodiscitis is *Staphylococcus aureus,* involved in over 40 to 67% of cases, and *Mycobacterium tuberculosis* is identified in almost one-third of spondylodiscitis cases [23]. Gram-negative bacteria, such as *Escherichia coli* and *Pseudomonas* can also be involved [24]. Unfortunately, around 21 to 34% of cases remain negative, with no pathogen identified throughout numerous attempts [25].

The objective of this study is to analyze the demographic and laboratory data, comorbidities, and the causative pathogens of spinal infections in patients surgically treated for spondylodiscitis.

## 2. Materials and Methods

### 2.1. Study Design

Our retrospective study screened all patients between May 2010 and December 2023, identifying a total of 1210 patients who underwent surgical treatment for spinal pathologies in “Foisor” Clinical Hospital of Orthopedics, Traumatology, and Osteoarticular TB. Out of this total, 98 patients were submitted to surgery for the treatment of spondylodiscitis. In six patients, a co-infection was established (two cases with an association of M.tb. and Gram-Positive cocci, two patients with an association of M.tb. and Gram-Negative bacilli, one association between *Klebsiella* and another Gram-Negative bacillus, and one association between positive and negative Gram-Negative bacilli), therefore, these patients were excluded from the study.

Inclusion criteria consisted of patients who reported to our ambulatory care unit for back pain, associated with imaging findings of spondylodiscitis and patients who were referred to our hospital either from their family doctor or from another hospital. All the patients included in this study benefited from surgery for spondylodiscitis. Patients with primary tumors or metastases were excluded from this study, as these require a more complex approach due to the nature of the disease and may potentially influence the outcome of the study through shorter life expectancy and worse overall health. Patients with prior surgery for infectious, degenerative, or malformative spine conditions were also excluded.

All patients agreed to participate in this study and signed an informed consent. The research was conducted by the ethical principles for medical research in accordance with the Declaration of Helsinki from 1964 and its later amendments. This study was approved by the “Foisor” Clinical Hospital of Orthopedics, Traumatology, and Osteoarticular TB Ethical Council with registration number 12211/4 November 2024.

### 2.2. Evaluation Before Admission to the Hospital

Before hospital admission, all patients had initially consulted either our ambulatory care unit or a distinct hospital and the diagnosis of spondylodiscitis was set based on clinical examination supported by imaging criteria identified on plain spine radiographs, CT, or MRI. A set of blood tests, along with a cardiac evaluation and treatment adjustment for other medical comorbidities were performed before hospital admission and any identified modifiable blood-test abnormality was corrected. Most patients are referred to our center from other hospitals to undergo surgical procedures. Given that a preoperative biopsy would only delay adequate treatment, all sample collections were gathered during surgery.

### 2.3. Evaluation After Admission to the Hospital

Our criteria for hospital admission of patients with spondylodiscitis were the presence of one or more of the following: (1) clinical symptoms of spondylodiscitis—back pain during activities that does not improve with rest, pain during movement, postural changes, with forward bending or straightening up; (2) presence of fever, night sweats, or other inflammatory signs; (3) neurological impairment; (4) presence of other medical comorbidities, and laboratory studies including the following; (5) elevated ESR—reference range 0–15 mm/h; (6) elevated CRP—reference range 0–0.3 mg/dL; (7) elevated WBC—reference range 4–9 × 10^9^/L; (8) elevated fibrinogen level—reference range 180–350 mg/dL; (9) imaging studies suggesting infectious spondylodiscitis including signs of disc space narrowing along with irregularities in the vertebral end plates or bony disruption on X-rays or CT scans, or the presence of infection and abscesses on MRI; (10) sepsis; (11) general symptoms such as anorexia and weight loss associated with back pain; (12) history of previous spine surgery.

Demographic data were collected including gender, age, body mass index (BMI), smoking status, presence of diabetes mellitus or chronic kidney disease, living in a rural or urban area, onset of spondylodiscitis, and history of the actual condition, such as previous spine surgeries or presence of a concurrent infectious disease (urinary tract infection, upper respiratory infection, gastrointestinal tract infection, skin or mucosal abscesses).

At admission, blood tests were drawn, and particular attention was paid to the following variables: WBC, ESR, CRP, fibrinogen level, and value of preoperative hemoglobin. Blood samples were drawn on vacutainers with different additives (none, K3 EDTA, sodium citrate) by a specialized healthcare assistant. The vacutainers were then labeled and transported to the in-hospital laboratory within 10–15 min. Blood cultures were not routinely performed and were drawn in patients who developed fever or chills under antibiotic treatment or had severe progression of the disease.

Patients were evaluated at admission by a spinal specialist using the Visual Analogue Scale (VAS) for back pain [26] and the Frankel classification used to assess neurological impairment [27]. Plain radiographs of the spine and MRI were performed centered on the affected levels. For cases in which an MRI could not be obtained a CT was performed. Screening tests for patients exposed to *Mycobacterium tuberculosis* were done in an external facility. As most patients are referred to their family physician or the National Tuberculosis Center, some patients may be lost to follow-up, therefore, data about mortality was not available.

### 2.4. Radiological Assessment

All patients are routinely submitted to anteroposterior (AP) and lateral (LL) spine radiographs in our Department of Radiology and Medical Imaging. All radiographs were performed on a DigitalDiagnost R3.1 machine (Philips Medical Systems Nederland B.V., Amsterdam, The Netherlands). Whenever the patient can stand, the spine radiographs are performed in a standing position. For the thoracic region of the spine, the anteroposterior view is performed with the patient erect, with hands placed along the sides, in arrested inspiration in order to push the diaphragm downwards, centered on the 7th thoracic vertebra. The central beam is perpendicular to the image receptor. Exposure parameters are 70–80 kVp and 40–70 mAs. The lateral view for the thoracic spine is performed with the patient erect, with humeri extended to 90 degrees, in suspended expiration, centered on the 7th thoracic vertebra. Exposure parameters are 80–100 kVp and 40–80 mAs. If the patient is unable to stand, a supine or lateral decubitus spine radiograph is performed. For the lumbar region, the anteroposterior view is performed with the patient erect, with hands placed along the sides, in suspended expiration and a posteroanterior position, which offers a lower radiation dose to the gonadal region. Exposure parameters are 70–80 kVp and 80–110 mAs. The lateral view is performed with the patient erect, with humeri extended to 90 degrees, in suspended expiration, in order to minimize the overlying of the diaphragm over the superior lumbar spine. Exposure parameters are 70–90 kVp and 60–100 mAs. If the patient is unable to stand, a supine or lateral decubitus spine radiograph is performed as well. All views are performed with an anti-diffusion X-ray grid and a focus-film distance of 115 cm. The digital images are verified for quality and then archived. All images were stored and accessed through the hospital picture archiving system and viewed using a dedicated radiology monitor and software. While X-ray examinations of the spine are often within the limits of normality in the early phases of the disease, complementary imagistic examinations, such as MRI or CT scans were also used [28]. The MRI and CT evaluations were performed in external facilities.

### 2.5. Laboratory Studies Performed

After arrival in the laboratory section of the hospital, the labeled vacutainers were split, and each was analyzed on the respective machine. For the complete blood count (CBC), blood samples were drawn on vacutainers containing K3 EDTA and analyzed on Siemens ADVIA^®^ 2120i (Siemens Healthineers, Erlangen, Germany). The ESR was drawn on vacutainers with sodium citrate 0.4 mL and measured manually using the Westergren technique. Biochemistry was drawn on vacutainers without any additives, and after centrifuging on Hettich ROTINA 35^®^, the Siemens Sysmex^®^ CA 600 analyzer (Siemens Healthineers, Erlangen, Germany) was used to perform the analysis.

The blood samples for coagulation tests, as well as fibrinogen, were drawn on vacutainers containing sodium citrate 2.7 mL, and after spinning on Hettich ROTINA 35^®^ (Andreas Hettich GmbH & Co. KG, Tuttlingen, Germany), it was then analyzed with the help of Siemens Sysmex^®^ CA 600 analyzer (Siemens Healthineers, Erlangen, Germany).

The CRP was analyzed and measured on Siemens Dimension^®^ (Siemens Healthineers, Erlangen, Germany).

### 2.6. Microbiological Analyses and Isolation of Bacteria

In order to identify the causative pathogens, samples for bacteriological analysis were taken from the affected site (affected disc, abscess, affected vertebra) either by a needle biopsy under fluoroscopic guidance or by direct biopsy during surgery. Antibiotics were always administered after the sample collection. All samples were divided into three groups, one used for Gram staining followed by cultures on different media pursued for 14 days, one used for Ziehl–Neelsen staining followed by cultures on Lowenstein–Jensen medium pursued for 60 days in a different facility, and the third sample for histopathological examination.

**Figure 1:**
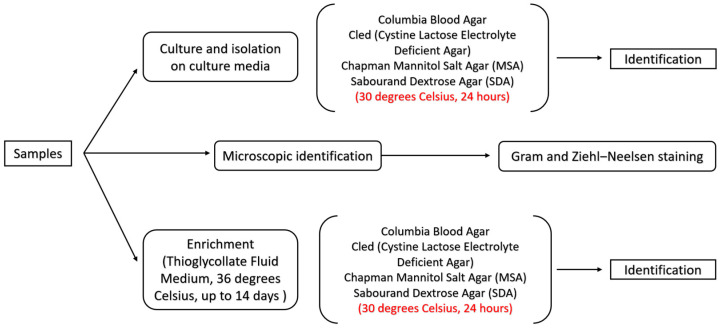
Schematic representation of the management of microbiological identification from patient samples. All samples are divided into three groups: one used for microscopic identification, one used for cultures on different culture media, and the third one used for enrichment.

The samples that arrived at the in-hospital laboratory were split into three groups: one used for microscopic identification with Gram staining, one used for culture and isolation on four different culture media, and the third one used for enrichment for up to 14 days (Figure 1).

The culture media used for culture and isolation were Columbia Blood Agar, CLED—Cystine-Lactose-Electrolyte-Deficient Agar, Chapman Mannitol Salt Agar, and Sabourand Dextrose Agar. After 24 h of incubation at 36° Celsius, a first microscopic identification is performed along with automatic identification using the Siemens Walk-Away 40 Plus^®^ (Siemens Healthineers, Erlangen, Germany). If cultures are positive, an antibiogram is performed using the Mueller–Hinton Agar with manual identification using the disk diffusion method and semi-automatic identification with the ErbaScan^®^ using the Minimum Inhibitory Concentration (MIC). All antibiograms are performed according to the latest recommendations by the European Committee on Antimicrobial Susceptibility Testing (EUCAST) [29].

**Figure 2:**
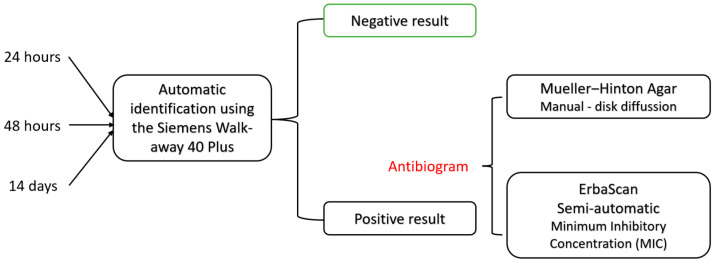
Schematic representation of the microbiological identification and results. An automatic identification of pathogens is used at 24 h, 48 h, and 14 days, followed by the antibiogram done manually with disk diffusion or semi-automatic with minimum inhibitory concentration, according to the latest recommendations by the European Committee on Antimicrobial Susceptibility Testing (EUCAST).

Provided that the first incubation and identification at 24 h is negative, a second microscopic identification from the enriched medium is performed at 48 h, following the same steps. If neither the first nor the second identification is positive, the samples are kept for enrichment in Thioglycollate Fluid Medium, at 36° Celsius for up to 14 days, with a third microscopic identification on day 14. Lastly, if the third attempt for bacterial identification remains negative, the final result will be negative (Figure 2).

### 2.7. Provided Treatment

All patients who underwent surgical treatment for spondylodiscitis signed informed consent. All cases were openly discussed during the routine daily morning meetings with the hospital orthopedic surgery staff. All patients benefited from surgery by an experienced spine surgical team based on individual case particularities. This could vary from fixation of at least two levels above and two levels below the affected region, along with decompression of the affected region and thorough debridement of infected tissues and evacuation of the various abscesses present, to combined anterior debridement and decompression and posterior fixation, whenever needed. As previously mentioned, samples for bacteriological analysis and histopathological examination were taken from the affected site during surgery. Antibiotic treatment was administered immediately after sample collection to ensure better microbiological analysis.

After surgery, all patients received empirical intravenous broad-spectrum antibiotics with Vancomycin 1 g/12 h and Cefuroxime 1.5 g/24 h. Antibiotics were always administered after the sample collection for the bacteriological and histopathological analysis. In the case of a known allergy to Penicillin, Cefuroxime is changed to Ciprofloxacin 500 mg/12 h or Levofloxacin 500 mg/24 h. In patients with known chronic kidney disease and impaired creatinine clearance, Vancomycin is substituted with Linezolid 600 mg/12 h and Cefuroxime is adjusted according to the estimated glomerular filtration rate. For patients with known autoimmune disorders, the preferred antibiotic therapy is Vancomycin 1 g/12 h and Ceftriaxone 2 g/24 h.

The antibiotic therapy is changed in accordance with the culture results and bacteria sensitivity or the results from histopathological examination, as soon as these are available. All patients received at least 2 weeks of intravenous antibiotic therapy during their hospital stay. On discharge, patients were shifted to customized oral antibiotics as per the culture results and maintained for at least another 4 weeks.

Patients with negative bacteriological findings followed a protocol consisting of 2 weeks of intravenous Vancomycin 1 g/12 h and Cefuroxime 1.5 g/24 h, followed by 4 weeks of oral antibiotic therapy consisting of Trimethoprim-Sulfamethoxazole 1440 mg/12 h and Rifampicin 300 mg/12 h.

The total duration of postoperative antibiotic management for all pyogenic spondylodiscitis cases was 6 weeks, customized or adjusted as required. All patients underwent complete bloodwork in order to assess blood, liver, and kidney function once every two weeks during the antibiotic therapy.

For patients with granulomatous inflammation on the histopathological examination, anti-tuberculous treatment was provided until the final results from the cultures were available and included a combination of Isoniazid 300 mg/24 h, Pyrazinamide 2000 mg/24 h, Ethambutol 1200 mg/24 h and Rifampicin 600 mg/24 h for at least 3 months with a daily administration program, followed by intermittent administration of Isoniazid and Rifampicin for another 6 to 9 months, depending on the clinical, laboratory, and radiological findings. During the anti-tuberculous treatment, each patient was closely monitored by the local tuberculosis center. Patients were evaluated using serial laboratory studies (WBC, ESR, CRP, Fibrinogen), clinical examination, and X-rays at 6 weeks, 12 weeks, 6 months, and 12 months.

### 2.8. Statistical Analysis

Statistical analysis of the data was performed using MedCalc^®^ Version 14.8.1 (MedCalc Software bvba, Ostend, Belgium). Continuous variables were tested for normality using the Shapiro–Wilk test. Comparisons across three or more groups with normally distributed data were performed using one-way ANOVA. Dichotomic variables were assessed across sub lots using Fisher’s exact test. For categorical variables and non-normally distributed continuous variables, the Kruskal–Wallis test was used; this test was also utilized for comparing the pain scores across subgroups. To quantify the effect size, eta squared was calculated for ANOVA results, epsilon squared was reported for Kruskal–Wallis tests and Cramér’s V was calculated for Chi-square tests. Statistical significance was considered for *p* values < 0.05. No adjustments for multiple comparisons were applied due to the exploratory nature of the study, focusing on identifying patterns for further investigation.

## 3. Results

The patients were divided according to their culture results as a negative result (29 patients), positive result with *Tuberculosis* (26 patients), and positive result with other pathological agents (37 patients). All patients from the *Mycobacterium tuberculosis* group had a positive culture with rifampin resistance testing. The identified pathological agents were 21 cases of Gram-Positive germs and 15 cases of Gram-Negative germs (Table 1). Of the 92 patients included in the study, the mean age at admission was 54.12 ± 12.95 years, ranging between 22 and 80 years. Extended patient demographics are available in Table 2.

**Figure 3:**
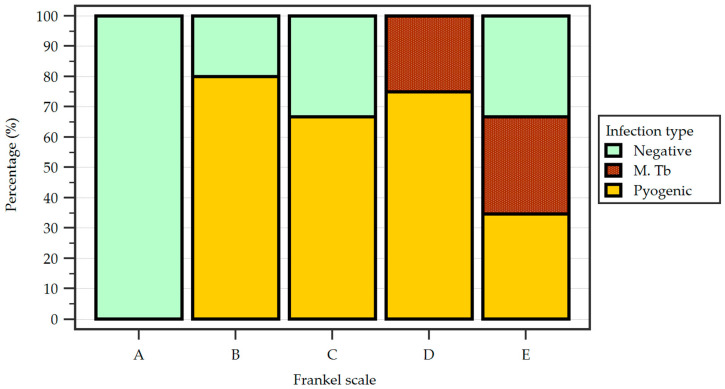
Frankel scale percentage distribution of results between the study groups.

There were no significant differences between the urban or rural area background and the type of pathological agent that determined the disease. Regarding the neurological impairment, the Frankel classification scale was used by the spinal specialist who evaluated all patients at admission, and the results were as follows: 79 patients were Frankel E, 5 patients were Frankel D, 3 patients were Frankel C, 5 patients were Frankel B and 1 patient was Frankel A, without any significant differences between the neurological impairment and the etiology of infection (Figure 3).

**Figure 4:**
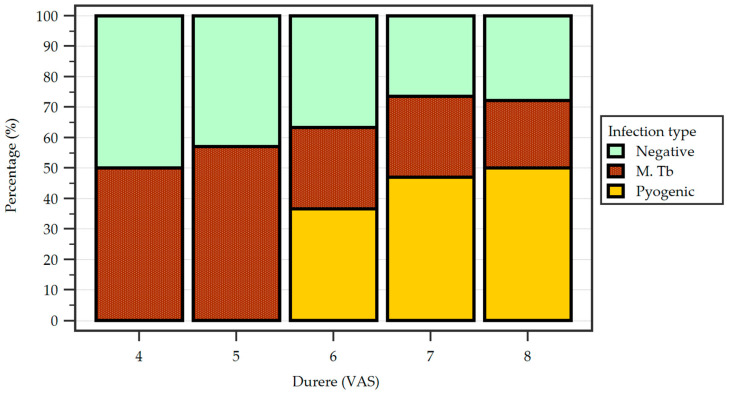
Reported pain according to the Visual Analogue Scale (VAS) between the study groups with percentage distribution.

The Visual Analogue Scale (VAS) was employed by the spinal specialist who evaluated patients at admission in order to assess pain levels in each patient (Figure 4).

The BMI showed significant differences (*p* = 0.022), with patients with *Tuberculosis* having lower values than pyogenic (*p* = 0.0150) or negative (*p* = 0.0293) cultures.

Age was also an important factor to consider, as most patients were young, active individuals. A significant difference was registered, with patients affected by *Mycobacterium tuberculosis* being younger than patients with pyogenic spondylodiscitis (*p* = 0.0150), as well as patients with negative cultures (*p* = 0.0466).

**Figure 5:**
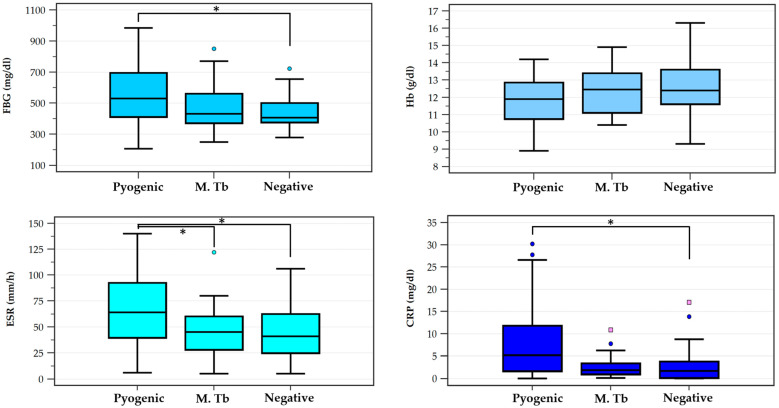
ESR, FBG, and Hb levels between the study groups. ESR = erythrocyte sedimentation rate, FBG = fibrinogen, Hb = hemoglobin. Significant differences (*p* < 0.05) between groups are marked with *. Circles represent outside values (beyond the lower/upper quartile ± 1.5 times the interquartile range); squares represent far out values (beyond the lower/upper quartile ± 3 times the interquartile range). Note: three extreme outliers were excluded from the CRP graph.

Following the thorough analysis of the complete blood work, several results showed significant differences. The WBC number registered no differences between groups. Significant differences were recorded in fibrinogen (*p* = 0.039) and CRP (0.009) levels between the negative cultures group and the pyogenic cultures group, with significantly lower levels in the first group. ESR level showed a significant difference (*p* = 0.042) between the pyogenic spondylodiscitis group and the other two groups, with higher levels of ESR registered in the first group. Although hemoglobin value showed no significant difference (*p* = 0.057) a trend between the positive cultures group and the negative cultures group was noted, with lower levels of Hemoglobin in the first group (Figure 5).

We also analyzed whether there are any differences in comorbidities and different types of pathogens involved in the etiology of spondylodiscitis. We looked at the smoking status of the patient (smoker or non-smoker), the presence of chronic kidney disease, and the presence of diabetes mellitus. Results showed a significant difference (*p* = 0.023) for patients with diabetes mellitus who were more prone to a tuberculosis etiology for their spondylodiscitis, and no significant differences between the smoker status or the presence of chronic kidney disease and the microbiological results for patients surgically treated for spondylodiscitis.

## 4. Discussion

Spondylodiscitis is an infectious disease with a massive impact on the healthcare system, affecting the socio-economical life of a patient [30]. As patients can be young and active individuals, early diagnosis and thorough adequate treatment are essential. The symptoms of spondylodiscitis can be non-specific, however, back pain of the affected region is the first one to appear, followed by neurological impairment at various periods of time depending on the first on-set of symptoms [26,31]. This can cause a delay in diagnosis, with pain increasing over time. In our study, a link between the levels of pain on the VAS scale at admission in the hospital and the etiology of the spondylodiscitis has been established, with patients that had pyogenic spondylodiscitis with positive cultures having higher levels of back pain than those with tuberculous spondylodiscitis and those with negative cultures.

*Mycobacterium tuberculosis* incidence is higher in developing countries, probably due to socio-economical imbalances and inequalities, with limited health resources for people who live in rural areas [32]. Among these, spinal tuberculosis is the most frequent extra-pulmonary form and can have various presentation forms, from back pain to severe cases of rapidly installed paraplegia [33]. Romania has a high incidence of infection with *Mycobacterium tuberculosis*, with recent studies showing incidence rates ranging from 58.41 to 689.32 per 100,000 inhabitants, with significant discrepancies around the country [34]. A prompt diagnosis is required to prevent massive destruction of the vertebral bodies with secondary mechanical instability of the spine. Although other causes for granulomatous inflammation can be present, in developing countries with a higher prevalence of *Mycobacterium tuberculosis* adequate treatment should be started as soon as possible. Purified protein derivative (PPD) skin tests, nucleic acid amplification tests, or interferon-gamma release assays testing are useful paraclinical examinations that can provide a rapid diagnosis for people exposed to *Mycobacterium tuberculosis* [35]. A limitation of our study was the inability to perform these tests, as they are usually carried out by the National Center for Tuberculosis, and were unavailable to us. Microbiological confirmation is obtained by culturing *Mycobacterium tuberculosis* from specimens obtained from the affected site, but these require three to eight weeks [36]. Due to the scarce presence of pathogens in the affected disc space and vertebral bodies, cultures may not become positive. While *Mycobacterium tuberculosis* infection is usually associated with migration and poverty [37,38], no patient in this study was a migrant.

Although conservative management with spinal immobilization associated with prolonged antibiotic therapy is the first line of treatment in patients without severe bony destruction [39], most patients referred to our hospital already had advanced bony destruction, with an indication for surgery. The spinal intervention could vary from fixation of at least two levels above and two levels below the affected region, along with decompression of the affected region and thorough debridement of infected tissues and evacuation of the various abscesses present, to combined anterior debridement and decompression and posterior fixation, whenever needed. During surgery, samples for bacteriological analysis and histopathological examination were taken from the affected site. Microbiological analysis was performed to ensure adequate and specific treatment for different pathogens involved in the etiology of spondylodiscitis, but also to assess antibiotic resistance. The most common pathogen isolated in the non-tuberculous cases was *Staphylococcus coagulase-positive*, followed by Gram-negative bacilli such as *Escherichia coli, Pseudomonas* spp., and *Proteus* spp., with frequencies similar to other reports [23,40,41].

The non-specific inflammatory laboratory work is useful for predicting the overall inflammatory status of the patient and predicting the presence of an infectious disease, such as infectious spondylodiscitis. Laboratory tests are usually non-specific, with elevated WBC, ESR, CRP, and fibrinogen. In our study, a significant difference was noted in the levels of ESR and fibrinogen between the different types of etiopathogenic agents of spondylodiscitis. The levels of serum WBC showed no difference between groups, which reinforces the findings of other studies that have proposed a limitation in the use of WBC as a predictor due to its low specificity and elevated levels in less than half of the patients [42]. ESR and CRP are more reliable due to their high sensitivity, with important use in the early diagnostic [43,44]. CRP yields a higher clinical value, as the levels correlate with the antibiotic treatment. As noted in our study, other findings included microcytic hypochromic anemia, correlation also found in different other papers [21].

Tuberculous spondylodiscitis is a chronic infection with non-specific inflammatory markers that can have normal or only slightly elevated levels compared to patients with acute pyogenic spondylodiscitis [45].

In order to obtain a larger and more complex perspective of the disease, several studies have compared different risk factors that can impact the disease, as well as their magnitude in the risk of disease determination. The presence of comorbidities was acknowledged as an important factor in morbidity and mortality rates [46]. The presence of different comorbidities in a patient and the effectiveness of their management plays an important role in infectious spondylodiscitis. General risk factors affecting the immune system, such as anti-inflammatory medication and autoimmune diseases heavily impact the disease course [47]. An important role is also played by the presence of diabetes mellitus, especially in cases with high fasting glycemia and high levels of glycate hemoglobin which indicate long periods of metabolic disruptions. The presence of chronic kidney disease is notable, as the renal system plays a crucial role in optimizing and good functioning of the immune system. In our study, 8 patients with chronic kidney disease and 20 patients with diabetes mellitus were registered. Among other pre-operative comorbidity factors, the presence of chronic pulmonary disease and a high updated Charlson Comorbidity Index score (uCCI) are thought to be independent risk factors [48,49]. Other comorbidity factors are congestive heart failure, rheumatologic, liver, and renal diseases, malignancy, dementia, immunosuppressant medication, AIDS/HIV, antiplatelet, or anticoagulant medication.

The BMI of patients has proven to be a very important factor in numerous infectious diseases alongside spondylodiscitis. BMI is a known predictive factor for periprosthetic joint infections, in fact being one of the risk factors that can be modified, with a huge impact on the risk infection of a patient [50]. BMI plays a crucial role in the significant rise of infection risk in all surgical interventions [51]. BMI can be modified through adequate diet and lifestyle modifications, and this change can determine a major decrease in the infectious risk. Overweight and obese patients have a three-times higher chance of unplanned revision surgery, a doubled rate of sepsis, and a higher rate of liver failure compared to normal-weight patients [52,53].

Smoking is associated with important systemic modifications, from minor blood vessels to entire organs, and smoking habits as well as nicotine quantity are in direct relationship with the immune system and the risk of infectious diseases [54]. Smoking is associated with higher levels of inflammatory markers [55].

Among the limitations of this study, we can include the retrospective recording of comorbidities without the possibility of extensive history of prior medical and pathological history. Retrospective studies may also carry potential inherent biases related to data collection, quality, and entry although we did not identify any compromised records in our study. Serum albumin being the richest protein in plasma, plays an important role in the defense system, including inactivation of toxic compounds, enzymatic and antioxidant properties, as well as offering protection in some toxic-mediated syndromes [56]. Another limitation of this study may be the impossibility of serum albumin dosage for all patients included in this study. The number of cases included in this study is relatively small due to the overall number of surgical procedures for spondylodiscitis. Multicenter studies might provide a broader view of the determination of patient outcomes and their relationship to etiopathogenesis and patient-specific factors.

## 5. Conclusions

Infectious spondylodiscitis remains a huge problem in healthcare and early diagnosis and prompt treatment are mandatory for the prevention of further destruction of spinal elements. Aggressive treatment might require more significant economic costs and involves significant mortality and potential antibiotic-resistance issues in the case of incorrect or incomplete treatment.

Risk factors and the presence of comorbidities should be explored with the intent of balancing patients as well as decreasing the associated morbidity and mortality. All modifiable risk factors should be addressed and adjusted. BMI is among the most important of all adjustable risk factors that can decrease the risk of unplanned revision surgery, sepsis, and liver failure. Smoking should also be ceased, or at least quantitively decreased.

Non-specific symptoms such as pain in the affected area of the spine and non-specific laboratory findings indicating a general inflammatory response may be identified in patients with infectious spondylodiscitis. The levels of WBC have limited use, due to low specificity and sensitivity. ESR and CRP are more reliable predictive factors, with higher sensitivity, and may be important in the early diagnosis. CRP is more sensitive, and it provides higher clinical value, as the levels correlate with the antibiotic treatment.

## Figures and Tables

**Table 1:** Results from cultures and identification of different pathological agents responsible for spondylodiscitis.

Result	Agent	Number of Cases
Negative results	-	29
Gram-Positive results	Cocci	20
	*Staphylococcus coagulase-positive*	*13*
	*Staphylococcus coagulase-negative*	*7*
	Bacilli	1
Gram-Negative results	Cocco-bacilli	1
	Bacilli	14
	*Proteus* spp.	*1*
	*Pseudomonas aeruginosa*	*3*
	*Escherichia coli*	*10*
Tuberculosis	*Mycobacterium tuberculosis*	26
Fungi	*Candida* spp.	1

**Table 2:** Demographic parameters and statistical significance of their difference across the study groups.

Parameter	Negative Cultures (n = 29)	Pyogenic Germs (n = 36)	M. tb. (n = 26)	*p* Value	Effect Size
Age (age)	56.48 ± 13.38	57.25 ± 10.99	49.04 ± 13.70	0.047 *	Ht = 6.1337, ε^2^ = 0.047
Sex (M/F)	23/6	21/15	12/14	**0.036** ‡	V = 0.191
BMI	28.02 ± 4.02	27.91 ± 3.53	25.24 ± 5.02	**0.022 †**	η^2^ = 0.083
Environment (rural/urban)	12/17	15/21	14/12	0.566 ‡	V = 0.079
Smoker (Y/N)	3/26	2/34	2/24	0.772 ‡	V = 0.053
Diabetes mellitus (Y/N)	11/18	5/31	3/23	**0.023 ‡**	V = 0.204
Chronic kidney disease (Y/N)	1/28	5/31	1/25	0.200 ‡	V = 0.133
VAS (4/5/6/7/8)	1/3/11/9/5	0/0/11/16/9	1/4/8/9/4	0.084 *	Ht = 4.9464, ε^2^ = 0.033
Frankel (A/B/C/D/E)	1/1/1/0/26	0/4/2/3/27	0/0/0/1/25	0.053 *	Ht = 5.8830, ε^2^ = 0.044
WBC (×10^3^/µL)	8.00 ± 1.91	8.35 ± 3.01	7.43 ± 2.44	0.629 *	Ht = 0.9256, ε^2^ = −0.012
CRP (mg/dL)	2.84 ± 4.04	10.23 ± 15.37	12.15 ± 41.23	0.009 *	Ht = 9.2429, ε^2^ = 0.082
Fibrinogen (mg/dL)	445.76 ± 108.15	554.41 ± 196.45	478.73 ± 153.53	0.039 *	Ht = 6.4408, ε^2^ = 0.050
ESR (mm/h)	46.66 ± 30.66	63.92 ± 32.57	46.15 ± 26.74	0.042 *	Ht = 6.3292, ε^2^ = 0.049
Hemoglobin (g/dL)	12.52 ± 1.62	11.69 ± 1.45	12.40 ± 1.37	0.057 †	η^2^ = 0.063

* Kruskal–Wallis test; † ANOVA; ‡ Chi-squared test; Ht = Kruskal–Wallis H-statistic corrected for ties; ε^2^ = epsilon squared; η^2^ = eta squared; V = Cramér’s V. M = male, F = female, Y = yes, N = no, BMI = body mass index, VAS = visual analog scale, WBC = White Blood Count, ESR = Erythrocyte Sedimentation Rate, CRP = C-Reactive Protein, M. tb. = Mycobacterium Tuberculosis. Bold values represent statistically significant results.

## Data Availability

The data presented in this study are available on reasonable request from the corresponding author.
